# Targeting Chemokines and Chemokine GPCRs to Enhance Strong Opioid Efficacy in Neuropathic Pain

**DOI:** 10.3390/life12030398

**Published:** 2022-03-09

**Authors:** Martina Vincenzi, Michele Stanislaw Milella, Ginevra D’Ottavio, Daniele Caprioli, Ingrid Reverte, Daniela Maftei

**Affiliations:** 1 Department of Physiology and Pharmacology “Vittorio Erspamer”, Sapienza University of Rome, 00185 Rome, Italy; daniela.maftei@uniroma1.it; 2 Toxicology and Poison Control Center Unit, Department of Emergency, Anesthesia and Critical Care, Policlinico Umberto I Hospital-Sapienza University of Rome, 00161 Rome, Italy; m.milella@policlinicoumberto1.it; 3 Santa Lucia Foundation (IRCCS Fondazione Santa Lucia), 00143 Rome, Italy; ginevra.dottavio@uniroma1.it (G.D.); daniele.caprioli@uniroma1.it (D.C.); 4 Laboratory Affiliated to Institute Pasteur Italia-Fondazione Cenci Bolognetti, Department of Physiology and Pharmacology “Vittorio Erspamer”, Sapienza University of Rome, 00185 Rome, Italy

**Keywords:** neuropathic pain, strong opioids, chemokines, chemokine receptors, GPCRs, chemokine receptor antagonist

## Abstract

Neuropathic pain (NP) originates from an injury or disease of the somatosensory nervous system. This heterogeneous origin and the possible association with other pathologies make the management of NP a real challenge. To date, there are no satisfactory treatments for this type of chronic pain. Even strong opioids, the gold-standard analgesics for nociceptive and cancer pain, display low efficacy and the paradoxical ability to exacerbate pain sensitivity in NP patients. Mounting evidence suggests that chemokine upregulation may be a common mechanism driving NP pathophysiology and chronic opioid use-related consequences (analgesic tolerance and hyperalgesia). Here, we first review preclinical studies on the role of chemokines and chemokine receptors in the development and maintenance of NP. Second, we examine the change in chemokine expression following chronic opioid use and the crosstalk between chemokine and opioid receptors. Then, we examine the effects of inhibiting specific chemokines or chemokine receptors as a strategy to increase opioid efficacy in NP. We conclude that strong opioids, along with drugs that block specific chemokine/chemokine receptor axis, might be the right compromise for a favorable risk/benefit ratio in NP management.

## 1. Introduction

**Figure 1:**
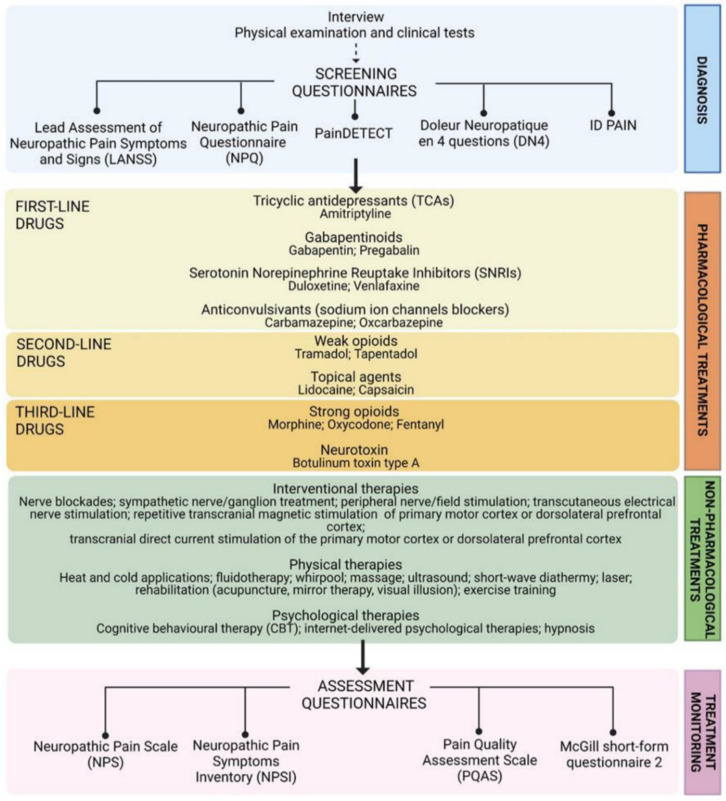
Scheme of the multidisciplinary approach for the management of neuropathic pain. Patient interview, physical and clinical tests and screening questionnaires-Leeds Assessment of Neuropathic Symptoms and Signs (LANSS) [14], Neuropathic Pain Questionnaire (NPQ) [15], painDETECT [16], Douleur Neuropathique en 4 Questions (DN4) [17], ID PAIN [18]—are the main steps of a comprehensive diagnosis [8] required to characterize neurological lesions, determine the presence of comorbidities, identify typical neuropathic symptoms and signs [19] and also assess the emotional, social, and economic impact of NP on patients’ lives [20]. Focusing on clinical symptoms rather than causative events/factors [2,10], NP treatment currently includes both pharmacological and non-pharmacological (interventional, physical, and psychological) options [8,9,10,21,22] whose efficacy and safety are constantly monitored with the following assessment questionnaires: Neuropathic Pain Scale (NPS) [23], Neuropathic Pain Symptoms Inventory (NPSI) [24], Pain Quality Assessment Scale (PQAS) [25], and the McGill short-form questionnaire 2 [26]. Among pharmacological options, according to the Grading of Recommendation, Assessment, Development and Evaluation (GRADE) system, gabapentinoids, tricyclic antidepressants (TCAs), serotonin-norepinephrine reuptake inhibitors (SNRIs), and anticonvulsants (sodium ion channel blockers) are strongly recommended for use and proposed by international guidelines as first-line drugs; weak opioids, lidocaine patches, and 8% capsaicin patches are weakly recommended for use and classified as second-line drugs; strong opioids and botulinum toxin type A have only weak GRADE recommendation and are relegate among third-line drugs.

Pain caused by a lesion or disease of the somatosensory nervous system is defined by the International Association for the Study of Pain (IASP) as neuropathic pain (NP) [1]. A wide range of etiological factors may contribute to the development of NP at the peripheral (peripheral neuropathic pain, PNP) or central level (central neuropathic pain, CNP) [2]. This heterogeneity and the widespread association with mood and/or sleep disorders [3], persistent inflammatory conditions and comorbidities [4,5,6] may cause underdiagnosing and undertreating neuropathic conditions [2,7]. Nowadays, the treatment of NP is a real challenge for physicians because the pharmacological tools available are not effective in many patients [8,9,10], and the mechanisms underlying the development and maintenance of NP are not fully understood [11,12]. Thus, a structured, stepwise, and multidisciplinary approach is recommended to improve NP diagnosis and treatment [13] (Figure 1).

The current available therapeutic recommendations for NP include both pharmacological (such as antidepressants, antiepileptics or opioids) and non-pharmacological approaches (such as interventional therapies or physical and psychological therapies) [8,9,10,21,22]. In this review, we focused on opioid therapy and refer readers elsewhere for more comprehensive reviews on alternative approaches [9,10,13,21,22].

Opioids, which are commonly prescribed and highly effective for the treatment of moderate to severe pain, are considered only a second or third choice for the treatment of NP [9]. This is because of their abuse potential, analgesia tolerance, and the often-reported exacerbation of pain sensitivity in patients that are already affected by NP [9,27].

Mounting evidence suggests that the mechanisms associated with opioid-induced hyperalgesia (OIH), a state of nociceptive sensitization caused by chronic exposure to opioids, have some commonalities with nerve injury-induced hypersensitivity [27]. It is therefore plausible that the mechanisms that promote OIH and NP might synergize and ultimately exacerbate NP [27,28] in NP patients treated with opioids.

In recent decades, numerous studies have indicated that peripheral nervous system (PNS) or central nervous system (CNS) damage leads to activation of glial cells, which leads to the release of pronociceptive mediators involved in peripheral and subsequent central sensitization [29,30,31,32]. Chemokines and chemokine receptors play a key role in the development and maintenance of the inflammatory components of NP [33,34]. Notably, increased expression of chemokines and chemokine receptors in neuronal and non-neuronal cells has also been described after opioid exposure [35]. The up-regulation of chemokines and the bidirectional interaction between chemokine receptors and opioid receptors (heterologous desensitization and heterodimerization) may therefore directly contribute to OIH and analgesic tolerance, and may explain the reduced efficacy of opioids in NP [36,37,38].

In this review, we first discuss the preclinical literature on the expression of various chemokines and chemokine receptors and their modulation in different animal models of NP. We then examine the changes in chemokine expression following opioid use in conditions with no neuropathic component and the crosstalk between chemokine and opioid receptors. Next, we review preclinical studies on the effects of inhibiting specific chemokines or chemokine receptors and how their blocking may increase opioid efficacy in NP. We conclude that antagonism of specific chemokines and chemokine receptors may not only improve the symptoms of NP per se but may also be clinically useful in increasing the efficacy of opioids in the treatment of NP. Pending the discovery of new and safe analgesics with innovative mechanisms of action, rational polytherapy currently seems to be the right compromise for a favorable risk/benefit ratio in the management of NP. 

## 2. Neuropathological Mechanisms Underlying Neuropathic Pain

NP occurs as a direct consequence of a disease or lesion of the somatosensory nervous system [1]. Depending on which part of the nervous system is primarily affected, NP can be divided into peripheral (due to lesions or diseases of the PNS) and central (due to lesions or diseases of the CNS) [39]. Patients experiencing NP may complain of intermittent or ongoing spontaneous pain, described as burning, shooting, tingling, pricking, pins and needles, or freezing pain, as well as stimulus-evoked types of pain [1,2]. In the last case, exposure to stimuli such as cold or light touch may manifest as either increased sensitivity to the painful stimuli, hyperalgesia, or pain triggered by non-painful stimuli, allodynia [1,2].

So far, the data from preclinical studies (using animal models that mimic the different aspects of human NP—see Table 1; we further refer readers to reviews [40,41]), have shown that NP symptoms are due to diverse anatomical, molecular, and electrophysiological changes which alter the normal sensory signaling from the periphery to the CNS [2]. 

These changes occur over the course of weeks or months and vary depending on the nature and extent of the disease or injury [104]. However, regardless of the aetiology, NP pathophysiology is characterized by a long-lasting and even permanent sensitization, both peripheral (through an increased stimulation response and a decrease in the nociceptive threshold of afferent nerve fibers [105]) and central (via increased responsiveness of nociceptive neurons in the spinal cord and CNS to their normal or subthreshold afferent inputs [1,106]).

A nerve injury, for instance, may thus increase the sensitization and excitability of primary sensory neurons, enhancing the excitatory synaptic transmission and decreasing the inhibitory transmission in CNS neurons [107,108]. The modulation of excitatory and inhibitory signaling has several neural correlates, such as the following: altered expression of ion channels (Na^+^, Ca^2+^, and K^+^) [109,110,111], up-regulation of certain neurotransmitters and neuromodulators such as substance P, calcitonin gene-related peptide, bradykinin, glutamate and neuropeptide Y, release of adenosine triphosphate, up-regulation of purinergic receptors, changes in protein kinase C and N-methyl-D-aspartate receptor (NMDA) activity, and changes in the expression of growth factors, cytokines, and chemokines [11,12,112,113].

In the last decade, it has been suggested that, in addition to changes in neuronal activity, the activity of non-neuronal cells, represented by immune cells (macrophages and lymphocytes) and glial cells (Schwann cells and satellite cells in the PNS, and microglia and astrocytes in the spinal cord and CNS), plays an important role in the regulation of NP [114,115,116,117,118]. Nerve damage leads, in fact, to the activation of resident macrophages and Schwann cells near the injury site, while satellite cells are activated in the dorsal root ganglia (DRG) [117,119]. Once activated, these cells begin to produce and release various proinflammatory mediators responsible for the recruitment of leukocytes to the site of injury, sensitization of sensory neurons, and activation of spinal glial cells [119]. On the other hand, sensory neurons and activated glial cells may also release proinflammatory factors that further sensitize neurons and lead to peripheral and central sensitization [120]. Activation of immune and glial cells in both the PNS and the CNS contributes to neuroinflammation by producing and releasing proinflammatory cytokines and chemokines, growth factors, and cellular adhesion molecules [31,32,34,121]. Therefore, it is important to understand the sequence and nature of the events underlying neuroimmune communication to find new mechanisms and targets for the treatment of NP.

In this regard, chemokines and chemokine receptors are gaining growing interest as modulators of neuronal plasticity and enhanced nociceptive transmission in NP conditions.

Chemokines are expressed and synthesized by immune and CNS cells under both physiological and pathological conditions [122,123,124]. They act as the key communication molecules between neurons, glial, and immune cells in pathological pain [33,34] and are involved in both peripheral and central sensitization following nerve injury [30,31,32]. Another main role of chemokines is to attract circulating granulocytes, lymphocytes, and monocytes to the site of injury, resulting in an inflammatory response [125]. Of note, it is now known that chemokines and chemokine receptors are expressed not only by immune cells but also by cells of the nervous system (e.g., neurons and glial cells) [122,126,127] (Box 1).

Box 1 **Chemokines and chemokine receptors.**

Chemokines, or ‘chemotactic cytokines’, are a family of small secreted (8–17 kDa) molecules that can induce directed chemotaxis of immune cells [128,129]. Most chemokines have two names, one referring to their biological activity, such as monocyte chemoattractant protein-1 (MCP-1) and the other to their structure [123]. Based on their structure and the position and number of conserved cysteine residues in the N-terminus, chemokines are classified into the four following subfamilies: CC, CXC, CX3C, and XC [130]. The CC-subfamily is the largest group of chemokines characterized by the adjacent positions of the first two of a total of four cysteine residues [131]. They contribute to a wide range of functions: they attract monocytes, eosinophils, basophils, T lymphocytes, natural killer (NK) cells, and dendritic cells [131,132]. The CXC-subfamily is the second largest group, characterized by a single amino acid separating the two cysteine residues, and is responsible for monocyte and granulocyte migration [133]. The XC-subfamily has only two closely related members (XCL1 and XCL2), characterized by two cysteine residues, and is responsible for the migration of lymphocytes but not neutrophils or monocytes. The CX3X-subfamily has only one member, CX3CL1 (also called fractalkine), which is characterized by three amino acids separating the two cysteine residues and acts as a chemoattractant and adhesion molecule for T lymphocytes, monocytes, and NK cells [119,131,132].

Chemokines exert their functions by activating G protein-coupled receptors (GPCRs) [134]. The nomenclature of chemokine receptors is CCR, CXCR, XCR, or CX3CR, analogous to their ligands. Chemokines within each subclass have promiscuous relationship with their receptors [134]: multiple chemokines can bind to the same receptor and a single chemokine can bind to multiple receptors. The exception to this rule is the interaction between CX3CL1 and its receptor, CX3CR1, which is monogamous [135].

In the case of peripheral nerve damage due to trauma, diseases, or drugs, a large number of chemokines and chemokine receptors are up-regulated in the PNS and/or CNS, and inhibition of these chemokines and chemokine receptors delays or alleviates pain behavior in the corresponding animal models [34,35,124,136]. For example, binding of the chemokine CCL2 (monocyte chemoattractant protein 1, MCP-1), particularly to the CCR2 receptor (C-C chemokine receptor type 2), has been reported to promote neuroinflammation and maintain the NP condition [137,138]. Up-regulation of CCL2 in primary sensory neurons of the DRG [138,139] and in neurons and astrocytes of the spinal cord [137,140] induces strong glial activation through CCR2 binding [141]. In addition, activation of CCR2 leads to activation of the p38MAPK pathway in microglia, resulting in the production of pronociceptive cytokines such as TNFα, IL-1β, IL-6, and IL-18 [30,121,137] and in spinal neurons, contributing to central sensitization through NMDA receptors [30]. Pharmacological or genetic inhibition of CCL2 and/or its receptor CCR2 alleviates pain in several animal models of NP [142,143] and inhibits glial cell activation [144].

CX3CL1 (fractalkine) has also been reported to be involved in the development and maintenance of NP and neuroinflammation [145]. These effects are mediated by the CX3CR1 receptor, whose expression has been shown to increase in spinal microglia after nerve injury [145]. Activation of CX3CR1 by CX3CL1 activates the microglial p38MAPK signaling pathway, leading to the production of pronociceptive molecules, i.e., IL-1β, IL-6, and TNFα [146]. The administration of neutralizing antibodies against CX3CL1 or CX3CR1 delays or attenuates chronic pain-related behaviors and reduces the release of proinflammatory factors [146,147], whereas CX3CR1 knock-out mice exhibit reduced pain behaviors correlated with decreased microglial activity [148].

Although CCL2 and CX3CL1 are the most extensively studied chemokines associated with NP, the roles of other chemokines and their receptors have been investigated in a variety of traumatic and non-traumatic NP conditions [124]. Of these chemokines, CCL1/CCR8, CCL3/CCR1, CCL3/CCR5, CCL4/CCR5, CCL5/CCR5, CCL7/CCR2, CCL21/CCR7, CXCL1/CXCR2, CXCL10/CXCR3, CXCL12/CXCR4, CXCL13/CXCR5, XCL1/XC1 expression has been shown to increase rapidly in primary sensory neurons and satellite cells of the DRG and in neurons and glial cells of the spinal cord, contributing to the development of hyperalgesia and allodynia via glial activation [124]. On the other hand, the inhibition of their functions by specific neutralizing antibodies, specific receptor antagonists, small interfering RNA (siRNA), or genetic ablation, leads to a marked reduction in NP and associated neuroinflammation [34,35,124,136].

Clinical studies analyzing the levels of different chemokines in the body fluids of patients with NP have shown that neuropathy increases the concentration of CX3CL1, CXCL5, CXCL10, CCL8, or CCL11 in cerebrospinal fluid (CSF) [149], CCL2, CCL3, CCL4, CCL19 in plasma [150,151], and CCL3, CCL4 in saliva [151]. Moreover, the high circulating levels of CCL2 revealed in patients with a traumatic spinal cord injury positively correlate with pain intensity [152].

A newly identified chemokine, prokineticin 2 (PK2), has been shown to play a critical role in the immune system and pain [153]. PK2 exerts its effects by activating two G-protein coupled receptors (GPCRs), prokineticin receptor 1 and 2 (PKR1 and 2), which are widely distributed in pain sites such as peripheral nerves, DRG, and the spinal cord [154]. PK2 and its receptors have been shown to be involved in the development and maintenance of experimental NP of various origins [155,156,157]. Indeed, neuropathy increases the expression of PK2 in Schwann cells, satellite cells, and primary sensory neurons of the DRG, astrocytes, and in the presynaptic terminals of the spinal cord [155,157]. On the contrary, blocking PK2 activity via specific PKR antagonists alleviates the neuropathy-induced pain and reduces the neuroinflammatory state in the sensory nerves, DRG, and spinal cord [155,156,157].

Despite the deleterious effects demonstrated for chemokines in NP, there are recent literature studies that suggest a neuroprotective role of some chemokines in other pathological conditions [158,159,160]. For example, the chemokine CCL5 acting on a GPCR named GPR75 (G Protein-coupled Receptor 75), which does not belong to the chemokine receptor family and is expressed by neuronal cells, activates intracellular signaling pathways associated with neuroprotective effects [159]. However, this CCL5/GPR75 neuroprotective effect has not been yet studied in NP conditions.

Considering their wide expression and activity on multiple regulatory pathways, modulation of chemokine/chemokine receptor signaling could represent a valuable target to develop new therapeutic options for NP.

## 3. Opioid Therapy and the Loss of Strong Opioid Analgesia in Neuropathic Pain

Opioids carry out their analgesic effects by modulating both the descending and ascending pain pathways [161,162], mainly through the activation of μ-opioid receptors (MORs) [163] and are therefore defined as MOR-agonists. Based on their binding affinity for MORs, opioids are classified as either weak or strong opioids. Although strong opioids such as morphine, oxycodone, and fentanyl are the most effective analgesics for the treatment of acute nociceptive pain and cancer pain [164], their usefulness in NP is still controversial due to use-related concerns (tolerance, OIH, abuse) [22,165,166] and the limited efficacy shown in clinical trials [167,168,169,170,171,172,173,174]. Surprisingly, despite the modest affinity for MORs, weak opioids such as tramadol and tapentadol have shown moderate efficacy and safety in various neuropathic conditions [175,176,177,178]. Some authors hypothesized that the better analgesic spectrum of the weak opioids over strong opioids in NP may be due to their dual mechanism of action, MOR-agonism, and noradrenaline/serotonin reuptake inhibition [179,180], whose contribution in antinociception depend on the type of pain treated [181].

Interestingly, the mechanisms behind the low efficacy of strong opioids in NP have not yet been elucidated, although strong opioid analgesia (using morphine as a reference molecule) has been characterized in several animal models of NP. These models have shown reduced drug efficacy in both allodynia [182,183,184,185,186,187,188,189,190,191] and hyperalgesia [182,185,189,190,192,193,194,195] and the development of analgesic tolerance following sustained or repeated opioid exposure. In addition, the administration of strong opioids may worsen rather than relieve NP [196,197,198,199,200]. Morphine treatment, in fact, may intensify the pre-existing allodynia in animals with peripheral injuries [200], and it may prolong allodynia for weeks to months after treatment has ended [196,197,198]. The same exacerbation of nociceptive hypersensitivity achieved with morphine is also achieved with oxycodone and fentanyl, as reported by Green-Fulgham et al. [199]. 

Some mechanistic explanations have been proposed for the loss of strong opioid effectiveness in neuropathic conditions. Among these, we can report the following: the down-regulation of MOR expression in the spinal cord [184,189] or DRG [188,193], increased methylation of the MOR gene promoter in primary sensory neurons [195], the release of peptides such as dynorphin [182,191] and cholecystokinin-B [186,194], the stimulation of glutamate receptors [195,201], the increase in serotonin levels [187] and a decrease in brain morphine concentration [190]. Moreover, as previously noted by Martinez-Navarro et al. [27], analgesic tolerance and OIH may share some mechanisms with NP, which are responsible for the failure of strong opioids in NP. The need for higher doses of morphine in NP, than in other pain conditions [189,202], and the exacerbation of nociceptive hypersensitivity when strong opioid dosing begins a few days to one month after nerve injury [198,199], support the lower sensitivity of NP to strong opioids and suggest a possible direct relationship between strong opioid analgesia and the type of pain treated. In this framework, neuro-inflammation following neuro-immune activation has been proposed as the common motif for the development and maintenance of opioid tolerance/hyperalgesia and NP [28,203]. Finally, the pro-inflammatory effect of opioids may be related to the up-regulation of cytokines and chemokines on neuronal and non-neuronal cells such as astrocytes, microglia, and immune cells [35,36].

## 4. Chemokine System as Novel Target for Enhancing Opioid Analgesia in Neuropathic Pain Therapy

A growing body of literature supports a critical role for chemokines in the development of opioid tolerance. For example, CCL2 levels are up-regulated by chronic opioid exposure in the dorsal spinal cord and CSF [204], astrocytes [205], human neurons [206], and human peripheral blood mononuclear cells [207]. In particular, chronic morphine exposure has been shown to enhance CCL2 immunoreactivity in the spinal cord, especially in spinal neurons, which was involved in morphine tolerance development [208,209]. The contribution of spinal neuronal CCL2 via CCR2 signaling to morphine tolerance was demonstrated by injection of a CCL2-neutralizing antibody. This approach resulted in significantly reduced antinociceptive tolerance and spinal microglial activation [208,209]. Moreover, the expression of other chemokines and their receptors, such as CXCL1/CXCR2, CXCL10/CXCR3, and CXCR12/CXCR4, is increased by opioids and seems to be associated with analgesic tolerance [210,211,212,213].

Instead, conflicting results, are available on the involvement of CX3CL1/CX3CR1 in opioid tolerance [214,215,216]. Johnston et al. [215] reported that CX3CL1 can modulate morphine analgesia through the release of interleukin-1 (IL-1) from the dorsal spinal cord. Thus, blocking CX3CR1 with a neutralizing antibody reduced the development of OIH and tolerance while enhancing morphine analgesia [215]. In contrast, Peng et al. [216] found that CX3CL1/CX3CR1 signaling in the spinal cord did not change with chronic morphine exposure, and neither a CX3CL1-neutralizing antibody nor a CX3CR1 antagonist could completely reverse the development of morphine tolerance. According to Peng and colleagues [216], Chen et al. [214] confirmed that the involvement of the CX3CL1/CX3CR1 axis in antinociceptive tolerance may be secondary to the effect of opioid administration on both glial activation and cellular localization of CX3CR1. Opioids may up-regulate the expression of CX3CR1, normally present on microglia, on neurons where CX3CR1 and opioid receptors may interact to form inactive heterodimers [214]. Although further studies are needed to define the role of heterodimers in painful conditions and opioid analgesic tolerance, some authors propose heterodimers as a new pharmacological target to enhance opioid analgesia [217,218,219,220]. For example, the administration of MCC22, a bivalent ligand (a MOR-agonist linked with a spacer to a CCR5-antagonist) of the MOR-CCR5 heteromer, in a mouse model of cisplatin-induced PNP leads to a reduction of hyperalgesia and spinal microglial inflammatory response without tolerance, reward, and alteration of motor function [218]. Comparable results were obtained by Akgün et al. [217] in LPS-treated mice.

In addition to chemokine release and heterodimerization, heterologous desensitization between chemokine- and opioid-receptors may also be involved in opioid tolerance [36]. Both the chemokine and opioid receptors are GPCRs (Box 2), and the activation of one of them by its ligand may promote the COOH-terminal phosphorylation-related inactivation of the other one (present on the same cell), with the loss of the ability to bind G-proteins, and thus activate the signal cascade [36]. No data are instead available on the influence of prokineticin system in opioid analgesia.

Box 2 **Focus on GPCRs.**

In GPCRs, seven transmembrane domains are linked by alternating intracellular and extracellular loops [221]. In the GPCR-inactive form, the cytoplasmic portion interacts with a heterotrimeric G protein formed by α, β, and ɣ subunits binding the GDP [222]. The agonist bound to the extracellular portion leads to a conformational change in the receptor and to the activation of one or more G-proteins [221,222] through the replacement of GDP with GTP [223]. The result is the dissociation of Gα from Gβɣ subunits [221]. Gβɣ dimer modulates several effectors (enzymes and ion channels), while Gα controls the receptor coupling specificity and the efficacy of Gβɣ modulation of ion channels [224,225,226]. Depending on their amino acid sequences, Gα subunits can be classified in four categories, Gα_s_, Gα_i/o_, Gα_q/11_, and Gα_12/13_ that differ for the downstream signaling pathway [223]. Generally, the Gα_s_ subfamily activates adenylyl cyclase, whereas the Gα_i_ subfamily inhibits adenylyl cyclase; the Gα_q_ subfamily activates phospholipase C; the Gα_12_ subfamily is involved in GTP-binding protein regulation [223]. Despite the fact that GPCRs do not share an overall identity in amino acids [227], they are classified into classes A, B, and C based on their sequence homologies [228]. Class A, also named “rhodopsin-like”, is the largest and most studied GPCR subfamily and includes receptors for rhodopsin, biogenic amines, and several peptide ligands. Class B includes receptors for hormones and neuropeptides such as vasoactive intestinal peptide, calcitonin, and glucagon, while class C consists mainly of metabotropic glutamate and γ-amino-butyric acid receptors and calcium receptors [228].

Both chemokine- and opioid-receptors are members of the class A GPCR family [228,229,230,231]. They are typically Gα_i/o_-coupled receptors [38], but the participation of other G-family members cannot be excluded [232,233,234]. They can activate several signal transduction pathways and lead to diverse responses. For example, both chemokine- and opioid-receptors may directly inhibit the activity of adenylyl cyclase leading to the reduction of intracellular cAMP [235,236]. They can also activate the mitogen-activated protein kinase cascade [38,134], especially the ERK1/2 [237,238] and p38 [239,240] pathways. The phosphoinositide-3-kinase and the following activation of NF-kB have also been described as a signal transduction pathway activated by both chemokines [241,242,243] and opioids [244,245]. Finally, chemokine- and opioid-receptors can stimulate phospholipase C to enhance the production of diacylglycerol and inositol 1,4,5-thriphosphate leading to an increase of the protein kinase C activity and of intracellular calcium levels, respectively [38,134].

**Figure 2:**
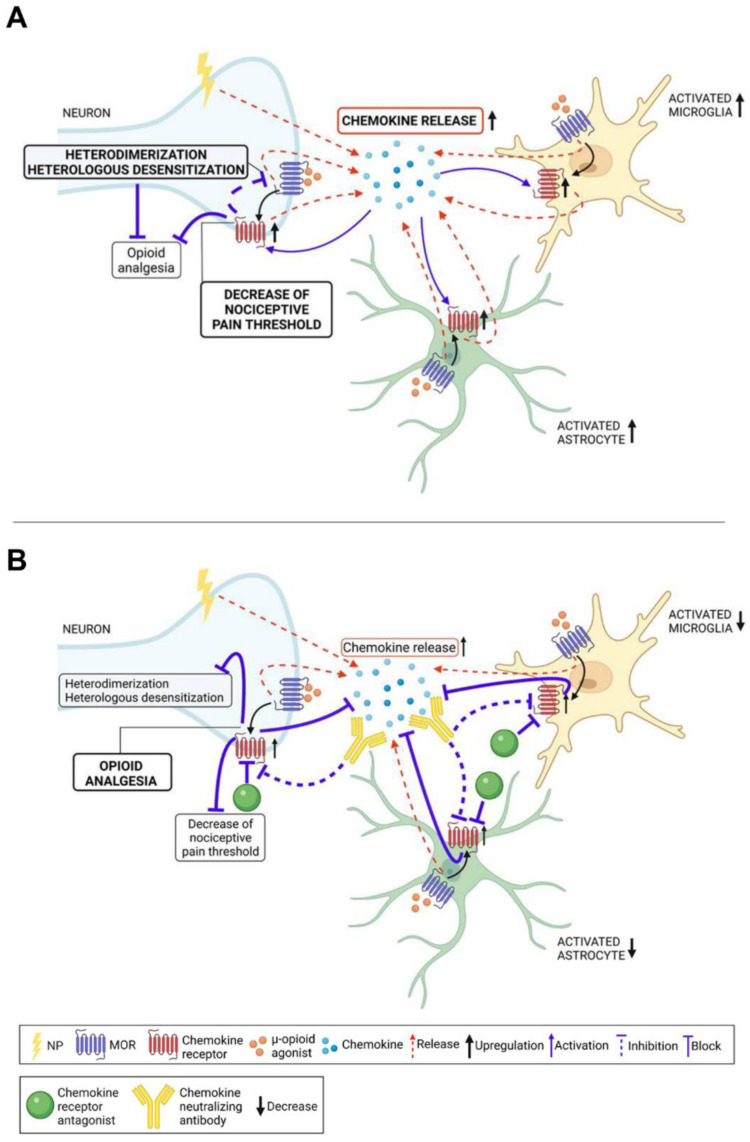
Involvement of chemokines and chemokine receptors in opioid-induced analgesia in NP. (**A**) Neuropathic pain conditions increase the production and release of a myriad of chemokines, whose binding to their specific receptors on neurons, astrocytes, and microglia decreases the nociceptive pain threshold and increases glial activation. Additionally, chronic μ-agonists administration further increases the chemokines and chemokine receptor expression and enhances the astrocyte and microglia reactivity in CNS. The neuronal activation of chemokine receptors by their specific ligands is followed by complex biochemical events that lead to heterodimer formation or heterologous desensitization between chemokine and opioid receptors, which lead to neuronal sensitization and reduce the opioid-induced analgesia. (**B**) The administration of chemokine-neutralizing specific antibodies or chemokine receptor specific antagonists reduces pain-related behaviors and glial cells activation and improve opioid efficacy in neuropathic conditions.

Given the involvement of chemokines in the development of both NP and opioid-tolerance/hyperalgesia, several researchers have hypothesized that administration of chemokine-neutralizing antibodies or their receptor antagonists may simultaneously reduce pain-related behaviors and improve opioid efficacy in neuropathic conditions [246,247,248,249,250] (Figure 2).

From the CC-subfamily, CCL1/CCR8, CCL2/CCR2-CCR4, CCL3-CCL4-CCL5/CCR5, CCL3-CCL9/CCR1, CCL7/CCR2, and CCL7-CCL11/CCR3 axes were investigated as novel pharmacological targets in several NP animal models. Noting that CCL1 administration induced mechanical and thermal hypersensitivity in naïve mice and that CCL1 levels in spinal neurons increased 7 days after streptozotocin (STZ) injection, Zychowska et al. [251] demonstrated that CCL1-neutralizing antibody administration reduces pain and improves the efficacy of morphine and buprenorphine in the STZ-diabetic neuropathy model. Similar results were obtained with CCL2- and CCL7-neutralizing antibodies in chronic constriction injury (CCI) mice [252]. The role of the CCL2-CCL7/CCR2 pathway in hypersensitivity and opioid effects has been demonstrated by injecting two different CCR2 antagonists, RS504393 and cenicriviroc, into CCI rats [248,253]. RS504393, a selective CCR2-antagonist, reduced pain-related behaviors and enhanced analgesia of morphine and buprenorphine by increasing mRNA and protein levels of pronociceptive (i.e., IL-1β, IL-18, IL-6, and inducible nitric oxide synthase, iNOS) and antinociceptive (i.e., IL-1α) factors [253], without affecting spinal expression of CCL2 and CCL7 [254]. Instead, cenicriviroc, a dual CCR2/CCR5 antagonist, alleviates mechanical/thermal hypersensitivity in CCI rats by decreasing the expression of CCL2, CCL7, and CCR2 in the spinal cord and CCR5 in the DRG and preventing the up-regulation of various pronociceptive mediators [248,254]. Cenicriviroc also improves the analgesic effect of morphine and buprenorphine, possibly by preventing the down-regulation of opioid receptors induced by the neuropathy in the DRG [248]. In addition, Bogacka et al. [255] reported that CCL2 can exert its pronociceptive effect by binding to CCR4. Thus, blocking CCR4 with its antagonist, C021, may reduce the development of mechanical and thermal hypersensitivity, improve the efficacy of morphine and buprenorphine, and stop the development of morphine tolerance in CCI mice [255].

A blockade of the CCL3-CCL4-CCL5/CCR5 axis with maraviroc, a CCR5 antagonist, also reduces NP symptoms by inhibiting the expression of CCL3, CCL4 and CCL5 and intensifies morphine and buprenorphine analgesia in the CCI neuropathy model [249]. The contribution of CCL3 and CCL9 through CCR1 signaling was investigated in the STZ-diabetic neuropathy model, and in CCI rats [250,256]. The CCL3- and CCL9-neutralizing antibodies and CCR1 antagonist (J113863) alleviated NP, and intensified the analgesic potency of morphine and buprenorphine [250,256]. Recently, the same effects of CCR1 antagonism were obtained by blocking CCR3 with its selective antagonist, SB328437 [257].

From the CXC-subfamily, CXCL10-CXCL11/CXCR3 and CXCR13/CXCR5 are proposed as novel therapeutic strategies to improve the efficacy of opioids in neuropathic diseases [258,259,260,261,262]. Ye et al. [262] found that the CXCL10/CXCR3 axis participates in cancer-induced bone pain (BCP) and that CXCL10, which is transiently up-regulated by morphine administration, can induce acute analgesia. Therefore, the inhibition of CXCL10 with a neutralizing antibody may enhance morphine analgesia [262,263]. CXCL11, another CXCR3 ligand, is up-regulated in spinal neurons and astrocytes during the development of morphine tolerance, and its inhibition by a neutralizing antibody may result in decreased morphine tolerance due to the blockage of the crosstalk between astrocytes and neurons [259]. In addition, Piotrowska et al. [260] suggested that blocking of CXCR3 with a selective antagonist, NBI-74330, could be a potential target to enhance the antinociceptive effect of morphine in CCI rats. Akin to CXCL11, CXCL13, which acts via CXCR5, appears to be involved in morphine tolerance in rats with cancer-induced bone pain [258,261]. Therefore, administration of a CXCR13-neutralizing antibody [261] or small interfering RNA (siRNA) of CXCR5 [258] may enhance morphine analgesia and prevent the development of tolerance. All the above-described novel target strategies to enhance strong opioid efficacy in neuropathic states are summarized in Table 2.

## 5. Conclusions

Neuropathic pain can be caused by a variety of insults to the peripheral or central somatosensory nervous system, including trauma, inflammation, ischemia, and metabolic or neoplastic disorders. The treatment of NP remains challenging as available analgesics (such as nonsteroidal anti-inflammatory drugs or opioids) fail to relieve pain due to lack of efficacy or serious side effects. The low efficacy of opioids on NP urges the demand for alternative therapeutic strategies. This has encouraged us to review the research on non-neuronal cells and proinflammatory mediators, such as chemokines, as a common mechanism contributing, on one hand, to the pathophysiology of NP, and on the other hand, to the development of opioid-related side effects. We suggest that inhibiting this pathogenic step by blocking chemokine production or chemokine receptor activity may be useful in alleviating the pain condition and increasing the efficacy of opioids. Pending the discovery of new and safe analgesics with innovative mechanisms of action, rational polytherapy currently seems to be the right compromise for a favorable risk/benefit ratio in the management of NP.

## Figures and Tables

**Table 1:** Human neuropathic pain and its corresponding animal model. Partial list of animal models used to mimic human neuropathic pain.

Categories	Human Neuropathic Pain	Type of Injury in Animal Models	Species
Central pain models	Thalamic syndrome (stroke in the thalamus)	Collagenase injection in ventral posterolateral nucleus of the thalamus [42]	Rats
	Mechanical injury to the spinal cord	Contusion or constant weight dropped over the spinal cord [43] Intraspinal injections of excitotoxins or excitatory amino acids [44,45] Injection of Erythrosin B (photosensitizing dye) through the tail vein followed by surgical exposure of vertebrae to an argon ion laser [46,47]	Rats, Mice
		Hemisection [48]	Rats
Peripheral pain models	Complete nerve lesion	Complete transection of sciatic nerve [49] Brachial plexus avulsion [50,51] Caudal trunk resection [52]	Rats, Mice
		Tibial and sural nerve transection [53]	Rats
	Partial nerve lesion	Sciatic nerve chronic constriction injury [54] Partial sciatic nerve ligation [55] Spinal nerve ligation [56,57] Axotomy of tibial nerve and/or common peroneal nerves and/or sural nerve [58,59] Partial injury of the saphenous nerve [60,61] Injection of Zymosan, HMG, and TNFα in the sciatic nerve [62,63,64,65] Implanting of polyethylene cuff around the common branch of the sciatic nerve [66,67]	Rats, Mice
		Freezing of the sciatic nerve with a cryoprobe [68] Laser irradiation [69]	Rats
		Ligation of common peroneal nerve [70]	Mice
	Trigeminal neuralgia	Chronic constriction injury of infraorbital trigeminal branch [71]	Rats
		Partial ligation of the infraorbital trigeminal branch [72]	Mice
	Orofacial pain	Injection of formalin into the temporomandibular joints [73] Subcutaneous injection of carrageenan over the right maxilla [74]	Rats, Mice
Disease-induced pain models	Multiple sclerosis	Induction of experimental autoimmune encephalomyelitis by immunization with myelin oligodendrocyte glycoprotein [75] Intracerebral inoculation of Theiler’s murine encephalomyelitis virus [76]	Mice
	Postherpetic neuralgia	Subcutaneous injection of cells infected with varicella zoster virus in the foot [77,78] Injection of herpes simplex virus in the skin of the hind paw [79] Administration of resiniferotoxin, TRPV-1 agonist, for depletion of capsaicin-sensitive afferents [80]	Rats, Mice
	HIV-associated sensory neuropathy	HIV-protein gp120 delivery in sciatic nerve [81]	Rats
		Transgenic animals expressing HIV-protein gp120 under a GFAP promoter [82]	Mice
	Cancer pain	Direct inoculation of compatible murine cancer cells [83,84] Direct inoculation of tumor cells [85,86]	Rats, Mice
	Diabetes	Administration of the pancreatic B-cell toxins streptozotocin [87,88] Administration of alloxan [89] Transgenic animals of type I and II diabetes [90,91,92,93]	Rats, Mice
Drug-induced neuropathy models	Anti-cancer agents-induced neuropathy	Administration of vincristine, cisplatin, oxaliplatin, or taxanes [94,95,96,97]	Rats, Mice, Guinea pigs
	Anti-HIV drugs-induced neuropathy	Administration of 2,3-dideoxycytidine [98]	Rabbits
		Administration of didanosine [99]	Rats
Inherited-induced pain models	Spontaneous neuropathy	Mutations of Trembler (Tr) and Trembler-J (Tr-J) in the PMP22 myelin gene [100] PMP22-transgenic animals [101,102] Mutations encoding for the myelin components P0 and connexin 32 [103]	Mice

**Table 2:** Novel target strategies that may increase the opioid analgesic efficacy in neuropathic pain.

Chemokine/ Chemokine Receptors Axis	Target	Chemokine/ Chemokine Receptor Inhibitors	Effects on Opioid Efficacy under Neuropathic Pain
CCL1/CCR8	CCL1	Neutralizing antibody	↑ Analgesic effects of morphine and buprenorphine (STZ, mice) [251]
CCL2/CCR2- CCR4	CCL2	Neutralizing antibody	↑ Analgesic effects of morphine and buprenorphine (CCI, mice) [252]
	CCR2	RS504393 (antagonist)	↑ Analgesic effects of morphine and buprenorphine (CCI, rats) [253]
		Cenicriviroc (antagonist)	↑ Analgesic effects of morphine and buprenorphine (CCI, rats) [248]
	CCR4	C021 (antagonist)	↑ Analgesic effects of morphine and buprenorphine (CCI, mice) and delays the development of morphine-induced tolerance [255]
CCL3- CCL4- CCL5/CCR5	CCR5	Maraviroc (antagonist)	↑ Analgesic effects of morphine and buprenorphine (CCI, rats) [249]
		Cenicriviroc (antagonist)	↑ Analgesic effects of morphine and buprenorphine (CCI, rats) [248]
CCL3- CCL9/CCR1	CCL3	Neutralizing antibody	↑ Analgesic effects of morphine (STZ, mice) [256]
	CCL9	Neutralizing antibody	↑ Analgesic effects of morphine (STZ, mice) [256]
	CCR1	J113863 (antagonist)	↑ Analgesic effects of morphine and buprenorphine (STZ, mice; CCI, rats) [250,256]
CCL7/CCR2	CCL7	Neutralizing antibody	↑ Analgesic effects of morphine and buprenorphine (CCI, mice) [252]
CCL7- CCL11/CCR3	CCR3	SB328437 (antagonist)	↑ Analgesic effects of morphine and buprenorphine (CCI, rats) [257]
CXCL4- CXCL9- CXCL10- CXCL11- CCL21/ CXCR3	CXCL10	Neutralizing antibody	↑ Analgesic effects of morphine (BCP, rats) [262]
	CXCL11	Neutralizing antibody	↑ Analgesic effects of morphine (BCP, rats) and attenuates morphine-induced tolerance [259]
	CXCR3	NBI-74330 (antagonist)	↓ Levels of CXCL4, CXCL9, CXCL10, CXCL11 and CCL21 in DRG and spinal cord (CCI, rats) ↑ Analgesic effects of morphine but not of buprenorphine [260]
CXCL13/ CXCR5	CXCR13	Neutralizing antibody	↑ Analgesic effects of morphine (BCP, rats) and prevents the development of morphine-induced tolerance [261]
	CXCR5	siRNA	↑ Analgesic effects of morphine (BCP, rats) [258]

Abbreviations: BPC-bone cancer pain; CCI-chronic constriction injury; STZ-streptozotocin diabetic neuropathy; siRNA-small interfering RNA.

## Data Availability

Not applicable.
